# The Core of Gut Life: *Firmicutes* Profile in Patients with Relapsing-Remitting Multiple Sclerosis

**DOI:** 10.3390/life11010055

**Published:** 2021-01-14

**Authors:** Madina Kozhieva, Natalia Naumova, Tatiana Alikina, Alexey Boyko, Valentin Vlassov, Marsel R. Kabilov

**Affiliations:** 1 Department of Neurology, Neurosurgery and Medical Genetics of the Pirogov Medical University, 117513 Moscow, Russia; kozhieva.m@fccps.ru; 2 Institute of Chemical Biology and Fundamental Medicine SB RAS, 630090 Novosibirsk, Russia; alikina@niboch.nsc.ru (T.A.); Valentin.Vlassov@niboch.nsc.ru (V.V.); kabilov@niboch.nsc.ru (M.R.K.); 3 Department of Neuroimmunology of the Federal Center of CVPI, 117513 Moscow, Russia; boiko.a@fccps.ru

**Keywords:** multiple sclerosis, relapsing-remitting course, fecal bacteriobiome, 16S rRNA gene amplicon sequencing

## Abstract

The multiple sclerosis (MS) incidence rate has been increasing in Russia, but the information about the gut bacteriobiome in the MS-afflicted patients is scarce. Using the Illumina MiSeq sequencing of 16S rRNA gene amplicons, we aimed to analyze the *Firmicutes* phylum and its taxa in a cohort of Moscow patients with relapsing-remitting MS, assessing the effects of age, BMI, disease modifying therapy (DMT), disability (EDSS), and gender. Among 1252 identified bacterial OTUs, 857 represented Firmicutes. The phylum was the most abundant also in sequence reads, overall averaging 74 ± 13%. The general linear model (GLM) analysis implicated *Firmicutes/Clostridia/Clostridiales/Lachospiraceae/Blautia/Blautia wexlerae* as increasing with BMI, and only *Lachospiraceae/Blautia/Blautia wexlerae* as increasing with age. A marked DMT-related decrease in *Firmicutes* was observed in females at the phylum, class (*Clostridia*), and order (*Clostridiales*) levels. The results of our study implicate DMT and gender as factors shaping the fecal *Firmicutes* assemblages. Together with the gender-dependent differential MS incidence growth rate in the country, the results suggest the likely involvement of gender-specific pathoecological mechanisms underlying the occurrence of the disease, switching between its phenotypes and response to disease-modifying therapies. Overall, the presented profile of *Firmicutes* can be used as a reference for more detailed research aimed at elucidating the contribution of this core phylum and its lower taxa into the etiology and progression of relapsing-remitting multiple sclerosis.

## 1. Introduction

The culture-independent identification of a plethora of gut microorganisms, which has been made possible by rapid advancements in methodology and instruments, has contributed to revealing the intimate relationship between the microbiota and the host, and by now there is no doubt that the gut microbiota significantly shapes human health [1,2,3]. Multiple sclerosis (MS) is one of the neurological disorders with increasing evidence implicating the gut microbiome as a key susceptibility factor [4,5,6]: MS patients have dysbiosis compared to healthy individuals [7,8,9], although the cause–effect relationship between gut microbiota dysbiosis and MS so far has not been unambiguously revealed [2,10,11]. Microbial involvement has been suggested as a cause of some chronic inflammatory diseases, including MS [12,13,14], and hence as a potential target for safer novel therapeutic strategies to treat the disease [7,15]. In the case of relapsing-remitting MS (RRMS), switching from relapse into a more prolonged remittance stage is a minimum. Such microbiota-based strategies need a better outline of the relationship between the microbiota and the host [16] in the RRMS-afflicted subjects in different regions of the world, elucidating the global picture.

The pathological and clinical symptoms of MS may vary widely [17], often presenting a challenge for correct diagnostics [18,19]. Presently, the relapsing-remitting course of the disease is the most common [5,20] and, consequently, the most studied [5,21]. The differences existing between the microbiomes of MS patients and healthy subjects were found to be exacerbated in chloroform-resistant, spore-forming bacteria, which primarily belong to the phylum *Firmicutes* (the *Bacilli* and *Clostridia* classes) [22], the core phylum in the human gut [23,24,25].

However, some studies included fecal samples from RRMS patients only in the remission phase [26,27]. The geographic origin of the surveyed population may have a greater impact on the composition of the gut microbiota than BMI or sex [23,28]. The MS prevalence and incidence rate has been steadily increasing in Russia [29]. Based on all these factors, we decided to aim our study at obtaining a picture of the gut 16S rRNA gene amplicon sequence abundance and the diversity of the *Firmicutes* phylum and its taxa in a cohort of Russian RRMS patients in remission or relapse, and at assessing the effects of body mass index, disease modifying therapy, disability assessment, and sex within the cohort.

## 2. Materials and Methods

### 2.1. Participants and Fecal Sample Collection

Sixty-four patients with remitting-relapsing MS course, as diagnosed by the MacDonald criteria [30], which have been validated in Russia [31], were recruited for the trial (Table 1). All the patients underwent clinical examination to assess their neurological status and disability according to the Expanded Disability Status Scale (EDSS) [32]. Some of the patients received disease-modifying therapy (DMT) as high-dose interferon-beta-1. No other medicines were administered, as they could have modified the gut microbiome [33]. The patients had no history of treatment with antibiotics at least for 3 months prior to feces sampling, as well as no probiotics and/or probiotics as special supplementation. All the patients were duly informed, gave their consent to the study, and signed the informed consent.

Fecal samples were collected in 10 mL sterile fecal specimen containers and stored frozen at approximately −20 °C. Samples were transferred to the laboratory within 1 week of collection and stored at −80 °C until they could be used for DNA extraction. The samples were collected at least one month prior to the corticosteroid treatment.

The protocol of the study was approved by the Ethics Committee of the Pirogov National Science and Research Medical University. All the clinical aspects of the study were supervised by a neurologist. New medicines, sorbents, and/or laxatives (including magnesium salts and castor oil), as well as any diet changes, were cancelled or not started at least one week prior to the fecal sample collection.

### 2.2. DNA Extraction and Sequencing

DNA was extracted from 50 to 100 mg of thawed patient fecal samples using the MetaHIT protocol [34]. The bead-beating was performed using TissueLyser II (Qiagen, Hilden, Germany) for 10 min at 30 Hz. No further purification of the DNA was needed. The quality of the DNA was assessed using agarose gel electrophoresis.

The 16S rRNA gene region was amplified with the primer pair V3–V4 combined with Illumina adapter sequences [35]. PCR amplification was performed as described earlier [36]. All the PCR reactions used 25 ng of fecal DNA as a template and were performed in triplicate. A total of 200 ng PCR product from each sample was pooled together and purified through MinElute Gel Extraction Kit (Qiagen, Hilden, Germany). The obtained libraries were sequenced with 2 × 300 bp paired-ends reagents on MiSeq (Illumina, San Diego, CA, USA) in SB RAS Genomics Core Facility (ICBFM SB RAS, Novosibirsk, Russia). The read data were deposited in GenBank under the study accession number PRJNA680445.

### 2.3. Bioinformatic and Statistical Analyses

Raw sequences were analyzed with the UPARSE pipeline [37] using Usearch v10.0. The UPARSE pipeline included the merging of paired reads; read quality filtering; length trimming; the merging of identical reads (dereplication); discarding singleton reads; removing chimeras; and operational taxonomic unit (OTU) clustering using the UPARSE-OTU algorithm. The OTU sequences were assigned a taxonomy using the SINTAX [38] and 16S RDP training set v16 [39].

The taxonomic structure of the obtained sequence assemblages—i.e., a collection of different species at one site at one time [40]—was estimated by the ratio of the number of taxon-specific sequence reads to the total number of sequence reads—i.e., by the relative abundance of taxa—which was expressed as a percentage.

The results are expressed as a mean and standard deviation (s.d.). Normal distribution was assessed using the Shapiro–Wilk test. A general linear model with two categorical factors (DMT and sex) and three continuous factors (age, BMI, and EDSS) was used to assess the influence of demographic and therapeutic variables on the *Firmicutes* abundance. Between-group comparisons were carried out as a post hoc analysis using the Fisher’s LSD test in the Statistica v.13.3 software (Statsoft, Tulsa, OK, USA). Alpha-biodiversity indices were calculated using Usearch.

## 3. Results

### 3.1. Total Bacteriobiome Diversity

After the quality filtering and chimera removal, a total of 1256 different OTUs were identified at a 97% sequence identity level, of which the overwhelming majority (1252) was *Bacteria*, with the other four representing the *Euryarchaeota* phylum of the *Archaea* domain.

Twelve bacterial phyla were identified, containing 21 classes, 25 orders, 54 families, and 174 genera, along with unidentified taxa. Most of the bacterial OTUs represented the *Firmicutes* phylum (857 OTUs, or ca. 68% of the total bacterial OTU number), with *Bacteroidetes* and *Actinobacteria* being the second and the third most OTU-rich phyla, with 148 (12%) and 84 OTUs (7%), respectively. *Clostridia* was the OTU-richest class (669 OTUs), accounting for 53% of the total OTU richness, with *Bacteroidia* (135 OTUs) and *Actinobacteria* (79) contributing 11% and 6%, respectively. Thus, these three classes drastically prevailed in the fecal bacteriobiomes studied.

Overall in the samples, the number of dominant OTUs—i.e., OTUs contributing ≥1% of the total sequence number in a sample—was 29, i.e., 2.0% of the total number of OTUs. They represented four phyla (*Firmicutes*, *Bacteroidetes*, *Actinobacteria*, and *Proteobacteria*), six classes (*Clostridia*, *Bacilli*, *Negativicutes*, *Bacteroidia*, *Actinobacteria*, and *Gammaproteobacteria*), 8 orders, 11 families, and 20 genera—i.e., far less taxonomic richness as compared to the total list of identified OTUs.

**Figure 1:**
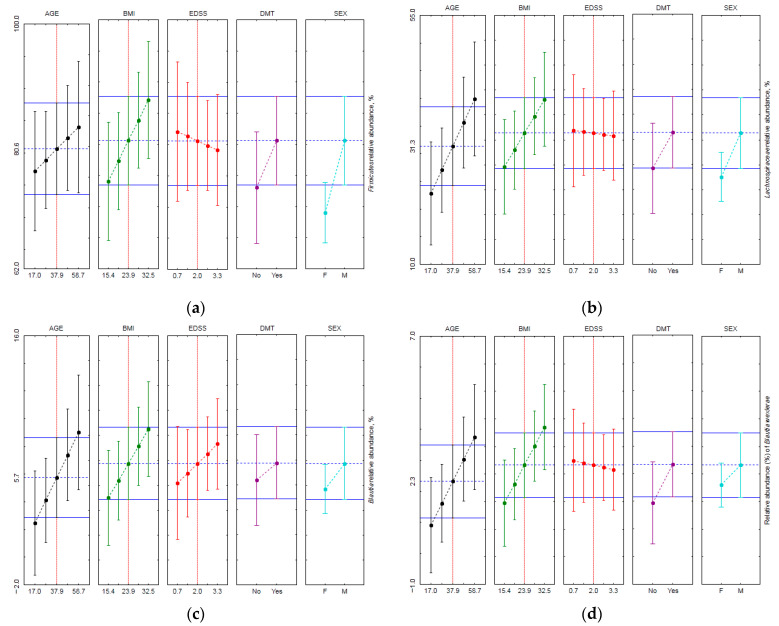
Profiles for the GLM-predicted values of relative abundance: (**a**) *Firmicutes*, (**b**) *Lachnospiraceae*, (**c**) *Blautia*, and (**d**) *Blautia wexlerae*. Markers show mean values and whiskers show 0.95 confidence intervals. DMT: No—no therapy. Yes—interferon treatment. Sex: F—females, M—males.

Members of the *Firmicutes* phylum were by far the most abundant not only in OTU richness but also in the relative abundance of sequence reads, which averaged 74 ± 13% for the studied cohort. The phylum was the only one with its relative abundance data complying with the normal distribution, as judged on the basis of Shapiro–Wilk’s test and the normal probability plots. Within the phylum’s lower taxonomic levels, *Clostridia*, *Clostridiales*, *Ruminococcaceae*, *Fecalibacterium*, and *Fecalibacterium prausnitzii* also showed a distribution pattern complying with the normal one. The performed statistical analysis, using GLM to elucidate the effect of age, body mass index, and EDSS as continuous factors (covariates) and sex and disease-modifying therapy as categorical factors, did not show age, BMI, and EDSS effects on *Ruminococcaceae* (Tables S4, S6 and S8) and its lower taxa, whereas the taxa *Lachospiraceae/Blautia/Blautia wexlerae* (Table 2; Tables S5, S7 and S9) had some age- and BMI-related shifts in their relative abundance. It should be noted that although the data on the relative abundance of *Lachospiraceae* and its *Blautia* genus and *Blautia wexlerae* were not normally distributed, their residuals were, therefore the GLM results pertaining to the taxa were taken into consideration. The profiles for the GLM-predicted relative abundance visualize these effects (Figure 1). The BMI effect in the studied cohort was revealed already at the phylum level (the predicted relative abundance is shown in Figure 1a), and further down at the class and order levels. The effect of sex and EDSS as individual factors was not statistically significant, with both making negligible contributions to the total data variance (Table 2).

### 3.2. DMT Effect on the Firmicutes Taxa Abundance

Disease-modifying therapy as a separate factor was found to have a negligible effect, both microbially and statistically, on the *Firmicutes* taxa abundance (Table 2, Tables S1–S9). However, quite a noticeable portion of the data variance was accounted for by the interaction between DMT and the patients’ sex (Table 2); a marked DMT-related decrease in *Firmicutes* was observed in females at the phylum, class, and order levels (Table 3, Tables S1–S3), and at the family level only for *Lachnospiraceae* (Table S5). At the same levels, there was a difference between females and males in the DMT subcohort, with males having substantially (more than 10%) increased relative abundance at the phylum, class, and order levels (Table 3) and the effect decreasing in its size and statistical significance at the family, genus, and species levels (*Lachnospiraceae/Blautia/Blautia wexlerae*).

The α-biodiversity indices calculated only for the *Firmicutes* OTUs were also subjected to GLM analysis and showed no DMT-related differential abundance (Table 4), averaging for the entire cohort 243 ± 56 observed OTUs (species richness), 299 ± 70 all OTUs (Chao-1), 3.51 ± 0.03 (Shannon), 0.145 ± 0.030 (evenness), and Fisher’s α (39 ± 10). However, the interaction between DMT and sex had an effect on the evenness, as DMT decreased this α-biodiversity index by 15% in males.

## 4. Discussion

Our study of the cohort of patients with relapsing-remitting MS found *Firmicutes* to be ultimately prevailing in the fecal bacteriobiome, accounting for more than three quarters of the relative abundance. In general, the finding agrees with the established global pattern of the phylum prevalence, albeit with some exceptions [41], in the gut/fecal bacteriobiome of both healthy individuals [24,42,43,44] and those compromised by various disorders/diseases [41,45,46]. In this study, we did not aim at comparing the RRMS cohort with healthy individuals, yet a lower percentage of *Firmicutes* was often reported for healthy cohorts elsewhere [26,42,47,48,49,50]. As for comparing our RRMS cohort with the RRMS cohorts of other ethnicity/regions, *Firmucutes* was at least 20% more abundant in the studied cohort as compared with the Italian and Japanese ones [26,51]; the effect very likely resulted from the stark national differences in diet between these cohorts.

The gut microbiota diversity is generally believed to change with age [44,52], and our cohort was not an exception, as age accounted for several percent of the variance in the *Lachnospiraceae* family, the second-ranked family in terms of relative abundance in the *Firmicutes* phylum; its *Blautia* genus; and *Blautia wexlerae*, one of the main dominant species in the study, with a ca. 2% abundance. Recently, a higher relative abundance of *Blautia* was reported in healthy Dutch adults as compared with children [53], and in children with cystic fibrosis *Blautia* was reported to be positively associated with age, the latter being the strongest predictor of overall fecal bacterial diversity [54]. However, we could not find reported association specifically between *Blautia*/*Blautia wexlerae* with the age of the MS-afflicted patients.

The *Firmicutes* abundance was reported to be associated with BMI in healthy individuals [55,56]. However, the information is sometimes confusing, as some studies reported increased BMI-related abundance [50,57], whereas others reported a decreased one [58]. As for the *Firmicutes* lower taxa, *Blautia*, for instance, was shown to be significantly and inversely associated with visceral fat accumulation by healthy Japanese people of both sexes [42]. Therefore, our finding that BMI accounts for several percent of the *Firmicutes* taxa abundance variance, with *Blautia/Blautia wexlerae* being mostly responsible for the effect (increase), apparently either complies or contradicts other reported data, in any case contributing to the general outline of RRMS bacteriobiome. Additionally, we could not find the information about *Blautia* abundance associated with BMI in other RRMS cohorts to make any comparison, so the implications of such association for disease phenotype and overall progression remains to be determined.

It is difficult to interpret changes in the *Blautia/Blautia wexlerae* presence in fecal assemblages in terms of the benefit for human health, as the reported data can be confusing or lacking. The overabundance of *Blautia* was reported in a Russian cohort with prediabetes and type 2 diabetes [57]; however, the genus was ultimately dominant also in healthy subjects with a normal level of glucose tolerance. On the other hand, some studies report *Blautia* as being proinflammatory [58].

Our finding that sex as a separate factor had not contributed to the data variance of the *Firmicutes* gut fecal assemblages of the RRMS patients may indicate the effect of some equalizing (in this respect) factor, since sex-related differences in the gut microbiota were revealed in some studies with autoimmune diseases [59].

Our result that disease-modifying therapy as an individual factor had not significantly contributed to the data variance was unexpected, as previous studies revealed DMT-related differences in the gut microbiota of MS patients: the dimethyl fumarate or interferon β-b treatment decreased the *Firmicutes* phylum relative abundance [60,61]. Yet, other studies reported an increased *Firmicutes* abundance, mostly driven by *Fecalibacterium*, in the dimethyl fumarate-treated patients [62], or no difference in the *Firmicutes* abundance between no-DMT and DMT cohorts [61]. However, in our study the observed 2% decrease in the *Fecalibacterium* abundance was not statistically significant and could hardly be physiologically relevant with its negligible contribution to the data variance. Our finding of sex-related differences in *Firmicutes* abundance between the no-DMT and DMT-subcohorts strongly suggests that there exists a sex-dependent effect on modulating the gut *Firmicutes* abundance according to DMT, at least in the studied RRMS cohort. The effect may be partially due to sex-associated diet differences [51,57,63,64], as the studied RRMS patients did not follow any specific diet recommendations.

The studied cohort included RRMS patients with clinical and neurodegenerative evidence of the two phenotypes of the disease activity—i.e., remission or relapse. However, the performed GLM analysis using EDSS as continuous factor, rather than using phenotype as a categorical one, showed that in the studied RRMS cohort there was no association between EDSS and the *Firmicutes* phylum and its lower taxa abundance. This is apparently in contrast with other studies that reported *Firmicutes* to be increased in MS patients during times of higher disease activity, and decreased at disease quiescence [26]. However, the discrepancy is very likely due to the absence of total synonymy between the EDSS and the clinical assessment of the disease activity phenotype, as well as other confounding factors—e.g., the timing and duration of DMT, etc.

*Fecalibacterium* representatives, and specifically *Fecalibacterium prausnitzii*, are known to metabolize dietary fibers as major short-chain fatty acid producers providing energy sources for enterocytes and achieving anti-inflammatory effects in the gut [65], and hence are generally considered to be beneficial for human health. The studied RRMS cohort overall had 11% of the genus relative abundance, thus being very close to the RRMS cohorts of some other ethnicities/races [66]. However, as the genus abundance showed no association with EDSS or DMT, it does not seem to be a promising target for microbiota modulation therapies to benefit RRMS patients’ condition.

Finally, we would reiterate some aspects pertaining to interpreting the fecal microbiome data in disease. Firstly, the overwhelming majority of the published results, based on the data for stool/feces samples, refer to such results euphemistically as the “gut microbiome” or the “intestinal microbiome”, rather than the “fecal microbiome”. However, as S. Fujio-Vejar et al. (2017) [42] rightly noted, the human fecal microbiota is not an entirely reliable reflection of the cecal or colonic microbiota. Secondly, a high inter-individual variability is observed in the studied populations/cohorts/groups, and our study is not an exception. The heterogeneity is usually attributed to an unspecified and hence unaccounted for plethora of influencing factors. However, one feces sample is usually collected from a sole stool event—i.e., without any individual replication. The latter, if performed, will decrease the intra-individual variability, consequently reducing some of the inter-individual one. Thirdly, we cannot help but note that associations between fecal/gut microbiota composition and host characteristics neither suggest nor prove any cause–effect dependence, allowing for two-way interpretation; the increased abundance of a certain taxon, especially apparently non-beneficial microbes, may be the essential enhancing agent of a disease, but it can also be one of the host’s means of adapting and/or mitigating some pathoecophysiological processes, triggered in a host by genetic and/or external factors. Last but not least, one should always bear in mind that the proportions of gene copy numbers, be they 16S rRNA or functional genes, are not entirely synonymic with the number of the relevant organisms present and the intensity of the processes they perform. Thus, the microbiome profiles provide just scaffolding that will be useful for constructing more comprehensive and/or targeted research.

## 5. Conclusions

Here, we presented the first gut bacteriobiome profiling of a Russian cohort of patients with relapsing-remitting multiple sclerosis, focusing solely on the *Firmicutes* phylum as the ultimate dominant and its taxa abundance as related to age, sex, body mass index, disease-modifying therapy, and disability assessment. The results of our study implicate sex as a modulating/driving factor in the fecal *Firmicutes* assemblages examined. In general, the results comply with the sex-dependent differential incidence growth rate in Russia, which suggests sex-specific peculiarities in the pathophysiology of the disease. Our results also suggest the likely involvement of sex-specific pathoecological mechanisms underlying the occurrence and spread of the disease, switching between its phenotypes and response to disease-modifying therapies. We believe that our findings make a small but important contribution to constructing a global picture of the intestinal microbiota diversity in relapsing-remitting MS patients. The presented profile of *Firmicutes* indicates the avenues for detailed microbiological and physiological research aimed at elucidating the contribution of this core phylum and its taxa into the etiology and progression of relapsing-remitting multiple sclerosis.

## Figures and Tables

**Table 1:** Demographics of the study cohort (N = 64).

	Mean	Median	Min	Max
Age, years	37.8	37.0	19.0	62.0
BMI ^$^, kg/m^2^	24.0	23.4	18.3	35.6
EDSS ^&^	2.2	2.0	1.0	4.5

^$^ BMI stands for body mass index; ^&^ EDSS stands for expanded disability status scale.

**Table 2:** General linear model results for the factors’ contribution to the variance in different taxa sequence reads’ relative abundance.

	Taxonomic Level								
	Phylum	Class	Order	Family		Genus		OTU	
Factor	*Firmicutes*	*Clostridia*	*Clostridiales*	*Ruminococcaceae*	*Lachnospiraceae*	*Fecalibacterium*	*Blautia*	*Fecalibacterium prausnitzii*	*Blautia wexlerae*
Age	1.5	2.5	2.2	1.6	8.6 *	0.0	**7.9**	0.0	**8.7**
BMI ^$^	**6.3**	**6.2**	**6.8**	0.5	**6.4**	1.4	**6.6**	1.3	**9.7**
EDSS ^&^	0.2	0.1	0.1	0.0	0.0	0.5	1.6	0.6	0.1
DMT ^#^	0.0	0.4	0.5	0.6	0.3	0.1	0.5	0.1	0.5
Sex	1.6	2.4	2.3	4.1	0.0	0.0	0.1	0.0	0.4
DMT × Sex	**7.9**	**8.7**	**7.4**	0.3	**9.0**	2.1	3.9	2.0	4.2
Error	79.6	75.5	76.5	89.3	74.9	93.6	76.7	93.7	75.1

^#^ DMT stands for disease-modifying therapy; ^$^ BMI stands for body mass index; ^&^ EDSS stands for expanded disability status scale. * Factors (GLM) with the *p*-values ≤ 0.05 are highlighted in bold, and the *p*-values of 0.05 ≤ 0.10 are underscored.

**Table 3:** Relative abundance (%) of *Firmicutes*-specific sequence reads.

	Factor Rate			Taxon Level								
			N	Phylum	Class	Order	Family		Genus		Species	
				*Firmicutes*	*Clostridia*	*Clostridiales*	*Ruminococcaceae*	*Lachnospiraceae*	*Fecalibacterium*	*Blautia*	*Fecalibacterium prausnitzii*	*Blautia wexlerae*
Entire cohort			64	74.0	66.0	65.0	31.0	27.3	11.0	5.1	10.9	1.8
DMT ^#^	No		25	76.0	69.1	68.3	32.9	29.0	12.1	5.1	12.0	1.6
	DMT		39	72.7	63.9	62.9	29.7	26.1	10.3	5.0	10.3	2.0
Sex	Females		44	72.4	63.8	62.8	29.5	26.9	10.8	5.1	10.7	1.9
	Males		20	77.5	70.7	69.8	34.2	28.1	11.4	5.0	11.4	1.7
**DMT** ×**·Sex**	**No**	**Females**	**17**	77.0 b *	**70.1 b**	**69.1 b**	32.0	31.3 b	12.6	5.8	12.5	1.9
	**No**	**Males**	**8**	**73.8 ab**	**67.0 ab**	**66.5 ab**	34.8	24.2 ab	11.0	3.6	11.0	1.0
	**DMT**	**Females**	**27**	**69.4 a**	**59.8 a**	**58.9 a**	27.9	24.1 a	9.7	4.6	9.7	1.8
	**DMT**	**Males**	**12**	**80.0 b**	**73.2 b**	**72.0 b**	33.7	30.7 ab	11.7	5.9	11.6	2.2

# DMT stands for disease-modifying therapy; * different letters indicate that the values are different at *p* ≤ 0.05 (Fisher’s LSD test); the factors and differences at *p* ≤ 0.05 and *p* ≤ 0.10 are highlighted in bold or underscored, respectively.

**Table 4:** Alpha-biodiversity indices for *Firmicutes* OTUs in the fecal samples of patients with relapsing-remitting multiple sclerosis.

	Factor Rate		N	Richness (OTUs)	Chao-1	Shannon	Evenness	Fisher’s α
Entire cohort			64	243	299	3.51	0.145	39
DMT ^#^	No		25	243	299	3.47	0.140	39
	DMT		39	243	299	3.54	0.149	39
Sex	Females		44	246	303	3.56	0.150	40
	Males		20	237	290	3.42	0.134	38
**DMT** × **Sex**	**No**	**Females**	**17**	246	303	3.49	0.141 ab*	40
	**No**	**Males**	**8**	247	303	3.60	**0.156 b**	40
	**DMT**	**Females**	**27**	239	291	3.44	**0.137 ab**	38
	**DMT**	**Males**	**12**	236	289	3.41	**0.133 a**	38

^#^ DMT stands for disease modifying therapy; * different letters indicate that the values are different at *p* ≤ 0.05 (Fisher’s LSD test); the factors and differences at *p* ≤ 0.05 are highlighted in bold.

## Data Availability

The read data were deposited in GenBank under the study accession number PRJNA680445, and are available at https://www.ncbi.nlm.nih.gov/bioproject/PRJNA680445/.

## Supplementary Materials

The following are available online at https://www.mdpi.com/2075-1729/11/1/55/s1: Table S1. GLM results for the *Firmicutes*-specific sequence reads abundance. Table S2. GLM results for the *Clostridia*-specific sequence reads abundance. Table S3. GLM results for the *Clostridiales*-specific sequence reads abundance. Table S4. GLM results for the *Ruminococcaceae*-specific sequence reads abundance. Table S5. GLM results for the *Lachnospiraceae*-specific sequence reads abundance. Table S6. GLM results for the *Faecalibacterium*-specific sequence reads abundance. Table S7. GLM results for the *Blautia*-specific sequence reads abundance. Table S8. GLM results for the *Faecalibacterium prausnitzii*-specific sequence reads abundance. Table S9. GLM results for the *Blautia wexlerae* specific sequence reads abundance.
