# Supplementary material for: The Core of Gut Life: Firmicutes Profile in Patients with Relapsing-Remitting Multiple Sclerosis
Source: Life (Basel). 2021 Jan 14;11(1):55. doi: 10.3390/life11010055 (PMC7828771; doi:10.3390/life11010055)
Supplement: Supplementary file 1 [file life-11-00055-s001.pdf]

life

Supplementary Tables

The Core of Gut Life: *Firmicutes* Profile in Patients with Relapsing-Remitting Multiple Sclerosis

Madina Kozhieva [1], Natalia Naumova [2,*], Tatiana Alikina [2], Alexey Boyko [3], Valentin Vlassov [2] and Marsel R. Kabilov [2]

[1] Department of Neurology, Neurosurgery and Medical Genetics of the Pirogov Medical University, 117513 Moscow, Russia; kozhieva.m@fccps.ru
[2] Institute of Chemical Biology and Fundamental Medicine SB RAS, Novosibirsk 630090, Russia; alikina@niboch.nsc.ru (T.A.); Valentin.Vlassov@niboch.nsc.ru (V.V.); kabilov@niboch.nsc.ru (M.R.K.)
[3] Department of Neuroimmunology of the Federal Center of CVPI, 17513 Moscow, Russia; boiko.a@fccps.ru
* Correspondence: naumova@niboch.nsc.ru or nnaumova@mail.ru

Table S1. GLM results for the *Firmicutes*-specific sequence reads abundance.

Factor	SS	d.f.	MS	F	*p*	%*
Intercept	3582	1	3582	25	0.000	34.8
Age	151	1	151	1	0.309	1.5
BMI	652	1	652	5	**0.037**	**6.3**
EDSS	24	1	24	0	0.684	0.2
DMT	3	1	3	0	0.883	0.0
Sex	160	1	160	1	0.296	1.6
DMT*Sex	814	1	814	6	**0.021**	**7.9**
Error	8183	57	144			79.6

*Factor contribution into the total variance of the sequence reads relative abundance; # DMT stands for disease modifying therapy; $ BMI stands for body mass index; & EDSS stands for expanded disability status scale. Note: factors with a p-value ≤0.05 are highlighted in bold.

Table S2. GLM results for the *Clostridia*-specific sequence reads abundance.

Factor	SS	d.f.	MS	F	*p*	%*
Intercept	1954	1	1954	10.6	0.002	14.0
Age	345	1	345	1.9	0.177	2.5
BMI	868	1	868	4.7	**0.034**	**6.2**
EDSS	17	1	17	0.1	0.766	0.1
DMT	56	1	56	0.3	0.584	0.4
Sex	329	1	329	1.8	0.187	2.4
DMT*Sex	1210	1	1210	6.6	**0.013**	**8.7**
Error	10513	57	184			75.5

*Factor contribution into the total variance of the sequence reads relative abundance; # DMT stands for disease modifying therapy; $ BMI stands for body mass index; & EDSS stands for expanded disability status scale. Note: factors with a p-value ≤0.05 are highlighted in bold.

Table S3. GLM results for the *Clostridiales*-specific sequence reads abundance.

Factor	SS	d.f.	MS	F	*p*	%*
Intercept	1734	1	1734	8.7	0.005	11.7
Age	328	1	328	1.6	0.205	2.2
BMI	1010	1	1010	5.1	**0.028**	**6.8**
EDSS	19	1	19	0.1	0.761	0.1
DMT	72	1	72	0.4	0.550	0.5
Sex	344	1	344	1.7	0.195	2.3
DMT*Sex	1102	1	1102	5.5	**0.022**	**7.4**
Error	11365	57	199			76.5

*Factor contribution into the total variance of the sequence reads relative abundance; # DMT stands for disease modifying therapy; $ BMI stands for body mass index; & EDSS stands for expanded disability status scale. Note: factors with a p-value ≤0.05 are highlighted in bold.

Table S4. GLM results for the *Ruminococcaceae*-specific sequence reads abundance.

Factor	SS	d.f.	MS	F	*p*	%*
Intercept	1450	1	1450	16.9	0.000	26.5
Age	86	1	86	1.0	0.322	1.6
BMI	27	1	27	0.3	0.578	0.5
EDSS	2	1	2	0.0	0.876	0.0
DMT	32	1	32	0.4	0.540	0.6
Sex	223	1	223	2.6	0.112	4.1
DMT*Sex	14	1	14	0.2	0.682	0.3
Error	4882	57	86			89.3

*Factor contribution into the total variance of the sequence reads relative abundance; # DMT stands for disease modifying therapy; $ BMI stands for body mass index; & EDSS stands for expanded disability status scale. Note: factors with a p-value ≤0.05 are highlighted in bold.

Table S5. GLM results for the *Lachnospiraceae*-specific sequence reads abundance.

Factor	SS	d.f.	MS	F	*p*	%*
Intercept	45	1	45	0.3	0.578	0.4
Age	950	1	950	6.5	**0.013**	**8.6**
BMI	703	1	703	4.8	**0.032**	**6.4**
EDSS	3	1	3	0.0	0.888	0.0
DMT	31	1	31	0.2	0.644	0.3
Sex	0	1	0	0.0	0.966	0.0
DMT*Sex	992	1	992	6.8	**0.011**	**9.0**
Error	8273	57	145			74.9

*Factor contribution into the total variance of the sequence reads relative abundance; # DMT stands for disease modifying therapy; $ BMI stands for body mass index; & EDSS stands for expanded disability status scale. Note: factors with a p-value ≤0.05 are highlighted in bold.

Table S6. GLM results for the *Faecalibacterium*-specific sequence reads abundance.

Factor	SS	d.f.	MS	F	*p*	%*
Intercept	109	1	109	3.1	0.082	5.2
Age	1	1	1	0.0	0.885	0.0
BMI	30	1	30	0.9	0.355	1.4
EDSS	11	1	11	0.3	0.567	0.5
DMT	2	1	2	0.1	0.794	0.1
Sex	0	1	0	0.0	0.971	0.0
DMT*Sex	43	1	43	1.3	0.268	2.1
Error	1974	57	35			93.6

*Factor contribution into the total variance of the sequence reads relative abundance; # DMT stands for disease modifying therapy; [$] BMI stands for body mass index; [&] EDSS stands for expanded disability status scale. Note: factors with a p-value ≤0.05 are highlighted in bold.

Table S7. GLM results for the *Blautia*-specific sequence reads abundance.

Factor	SS	d.f.	MS	F	*p*	%*
Intercept	151		151	6.4	0.014	8.6
Age	138		138	5.9	**0.019**	**7.9**
BMI	116		116	4.9	**0.031**	**6.6**
EDSS	28		28	1.2	0.283	1.6
DMT	9		9	0.4	0.533	0.5
Sex	1		1	0.1	0.823	0.1
DMT*Sex	69		69	2.9	0.094	3.9
Error	1346		24			76.7

*Factor contribution into the total variance of the sequence reads relative abundance; # DMT stands for disease modifying therapy; [$] BMI stands for body mass index; [&] EDSS stands for expanded disability status scale. Note: factors with a p-value ≤0.05 are highlighted in bold, and factors with a p-value of 0.05 ≤0.10 are underscored.

Table S8. GLM results for the *Faecalibacterium prausnitzii*-specific sequence reads abundance.

Factor	SS	d.f.	MS	F	*p*	%*
Intercept	112	1	112	3,3	0,075	5,4
Age	1	1	1	0,0	0,885	0,0
BMI	27	1	27	0,8	0,377	1,3
EDSS	12	1	12	0,3	0,560	0,6
DMT	3	1	3	0,1	0,786	0,1
Sex	0	1	0	0,0	0,953	0,0
DMT*Sex	43	1	43	1,2	0,269	2,0
Error	1952	57	34			93,7

*Factor contribution into the total variance of the sequence reads relative abundance; # DMT stands for disease modifying therapy; [$] BMI stands for body mass index; [&] EDSS stands for expanded disability status scale. Note: factors with a p-value ≤0.05 are highlighted in bold.

Table S9. GLM results for the *Blautia wexlerae* specific sequence reads abundance.

Factor	SS	d.f.	MS	F	*p*	%*
Intercept	25	1	24.8	6.3	0.015	8.3
Age	26	1	26.1	6.6	**0.013**	**8.7**
BMI	29	1	28.9	7.3	**0.009**	**9.7**
EDSS	0	1	0.3	0.1	0.775	0.1
DMT	1	1	1.4	0.4	0.549	0.5
Sex	1	1	1.1	0.3	0.606	0.4
DMT*Sex	13	1	12.6	3.2	<u>0.078</u>	<u>4.2</u>
Error	224	57	3.9			75.1

*Factor contribution into the total variance of the sequence reads relative abundance; # DMT stands for disease modifying therapy; [$] BMI stands for body mass index; [&] EDSS stands for expanded disability status scale. Note: factors with a p-value ≤0.05 are highlighted in bold, and factors with a p-value of 0.05 ≤0.10 are underscored.
